# Gut microbial culturomics identifies autism-associated *Shigella* and reveals species-level remodeling during fecal microbiota transplantation

**DOI:** 10.1128/spectrum.00797-26

**Published:** 2026-06-04

**Authors:** Bin Chen, Zhenzhen Su, Yuqing Sun, Zhuofan Shao, Xiaoxiao Yu, Xin Jiang, Xinxin Xue, Lianhu Yu, Lihong Wang, Wenwen Zhao, Yuling Feng, Ke Ning, Meilu Zhang, Aihua Cao, Lei Zhang

**Affiliations:** 1 Microbiome-X, School of Public Health, Cheeloo College of Medicine, Shandong University 66555, Jinan, China; 2 Department of Pediatrics, Shandong University Qilu Hospital 91623, Jinan, China; 3 Department of Psychology, University of California 360715, Santa Cruz, California, USA; 4 School of Pharmaceutical Engineering, Jining Medical University 74496, Jining, China; Nanchang University, Nanchang, Jiangxi, China

**Keywords:** autism, microbiota, culturomics, fecal microbiota transplantation

## Abstract

Autism spectrum disorder (ASD) has been repeatedly linked to gut microbiota alterations, yet mechanistic insight remains limited by the scarcity of ASD-specific cultured isolates. Here, we performed large-scale gut microbial culturomics on fecal samples from 41 children with ASD and 12 typically developing (TD) controls, generating 1,724 isolates across six phyla. Longitudinal culturomics profiling was further conducted in 17 ASD children undergoing a 9-week fecal microbiota transplantation (FMT) intervention. All isolates underwent 16S rRNA sequencing and non-redundant clustering to assess species-level diversity, ASD-TD differences, and microbial dynamics associated with clinical response. ASD children harbored a distinct culturable microbiota enriched for *Shigella flexneri* and *Shigella boydii*, whereas TD children were enriched in beneficial taxa, such as *Bifidobacterium catenulatum* subsp. and other health-associated species. Notably, 20 species isolated from ASD children and 20 from TD children were absent from major existing gut microbiota biobanks, thereby expanding the cultivable repertoire. Among FMT participants, clinical responders exhibited increased alpha diversity, progressive enrichment of TD-associated beneficial taxa, such as *Bacteroides fragilis*, *Anaerostipes hadrus*, *Parabacteroides merdae*, and *Turicibacter sanguinis*, and a marked reduction of ASD-associated *Shigella flexneri* and *Shigella boydii*, whereas non-responders showed minimal shifts. Acquisition of TD-enriched strains at week 9 was strongly correlated with clinical improvement, suggesting that species-level ecosystem remodeling may contribute to FMT efficacy. This work establishes one of the first ASD-focused gut microbial culturomics resources, identifies *Shigella* as a potential ASD-associated taxon, and provides foundational evidence and testable mechanistic hypotheses for future microbiome-based interventions in ASD.

**IMPORTANCE:**

Most autism spectrum disorder (ASD) microbiome studies rely on sequencing, which identifies associations but lacks live strains needed for mechanistic tests. We cultured 1,724 isolates from ASD and typically developing (TD) children, providing an ASD-focused, strain-level resource. ASD samples showed a significantly higher prevalence of *Shigella flexneri*. Longitudinal profiling during fecal microbiota transplantation (FMT) showed that clinical responders gained TD-enriched taxa and lost *Shigella* spp., and these shifts correlated with symptom improvement. This resource enables functional assays and gnotobiotic studies with ASD-relevant strains and provides a foundation for rational microbiome-based interventions.

## INTRODUCTION

Autism spectrum disorder (ASD) is a neurodevelopmental disorder characterized by impaired social communication and repetitive behaviors (1). In China, ASD affects ~7‰ of children aged 6–12, posing significant challenges to families and public health (2). While behavioral interventions remain the mainstay of treatment, no pharmacological therapies are currently available to address core ASD symptoms (2, 3).

Emerging evidence has implicated the gut microbiota in ASD pathogenesis through immune, metabolic, and gut-brain axis pathways (4–10). Compared with typically developing (TD) individuals, children with ASD frequently exhibit altered gut microbiota composition and diversity (11), and fecal microbiota transplantation (FMT) has shown preliminary efficacy in improving gastrointestinal and behavioral symptoms. These findings highlight the gut microbiota as a promising therapeutic target for ASD (12).

Nevertheless, most ASD microbiome studies have relied on high-throughput sequencing approaches (13–15). While powerful in identifying microbial associations, sequencing alone cannot provide mechanistic insights due to the absence of live, culturable bacterial resources. Culturomics, the large-scale culture and isolation of gut microbes, offers a crucial complement by generating species-level resources for functional studies and causal investigations. Existing microbial culture collections, including the Broad Institute-OpenBiome Microbiome Library (BIO-ML) (16), the Culturable Genome Reference (17), the Human Gastrointestinal Bacteria Culture Collection (18), the human Gut Microbial Biobank (hGMB) (19), and the Human Intestinal Bacteria Collection (20), have significantly expanded our understanding of the human gut microbiome. However, ASD-specific microbial isolates remain largely absent from these repositories.

Here, we conducted a culturomics study of fecal samples from 41 ASD and 12 TD children. We successfully isolated 1,724 bacterial strains, thereby expanding the culturable microbiota repertoire. Moreover, by integrating longitudinal culturomics profiling in ASD patients undergoing FMT, we investigated species-level dynamics during treatment and identified candidate taxa, such as *Shigella flexneri* and *Shigella boydii*, that may be involved in ASD pathogenesis. This work establishes a foundational resource for functional microbiota studies in ASD and provides new insights into microbial contributions to treatment efficacy.

## RESULTS

### Culturomics reveals distinct gut microbiota composition and potential biomarkers in ASD and TD children

A total of 41 ASD children (37 males and 4 females) and 12 TD children (all males) were enrolled in this study. Participant characteristics are summarized in Table 1.

**TABLE 1:** Participants characteristics^*a*^

	Children with ASD (*n* = 41)	Children with TD (*n* = 12)
Gender		
Male	37 (90.24%)	12 (100%)
Female	4 (9.76%)	0 (0%)
Baseline characteristics		
Age	5.87 (1.42)	6.40 (1.80)
BMI	17.27 (5.54)	15.60 (1.70)
Scale score		
ADOS-2	23.63 (3.68)	NA
SRS	139.25 (19.99)	NA
CARS	35.44 (3.88)	NA
ABC	76.86 (31.68)	NA
Bristol stool score	3.07 (1.14)	NA
GSRS	22.96 (6.07)	NA
CGI-I	3.04 (0.94)	NA

^*a*^ Data for continuous variables are presented as mean (SD), and categorical variables are presented as *n* (%). ADOS-2, Autism Diagnostic Observation Schedule, second edition; SRS, social responsiveness scale; CARS, childhood autism rating scale; ABC, autism behavior checklist; GSRS, gastrointestinal symptom rating scale; CGI-I, clinical global impressions scale-improvement; NA, not applicable, as these clinical scales were only administered to children with ASD.

**Fig 1:**
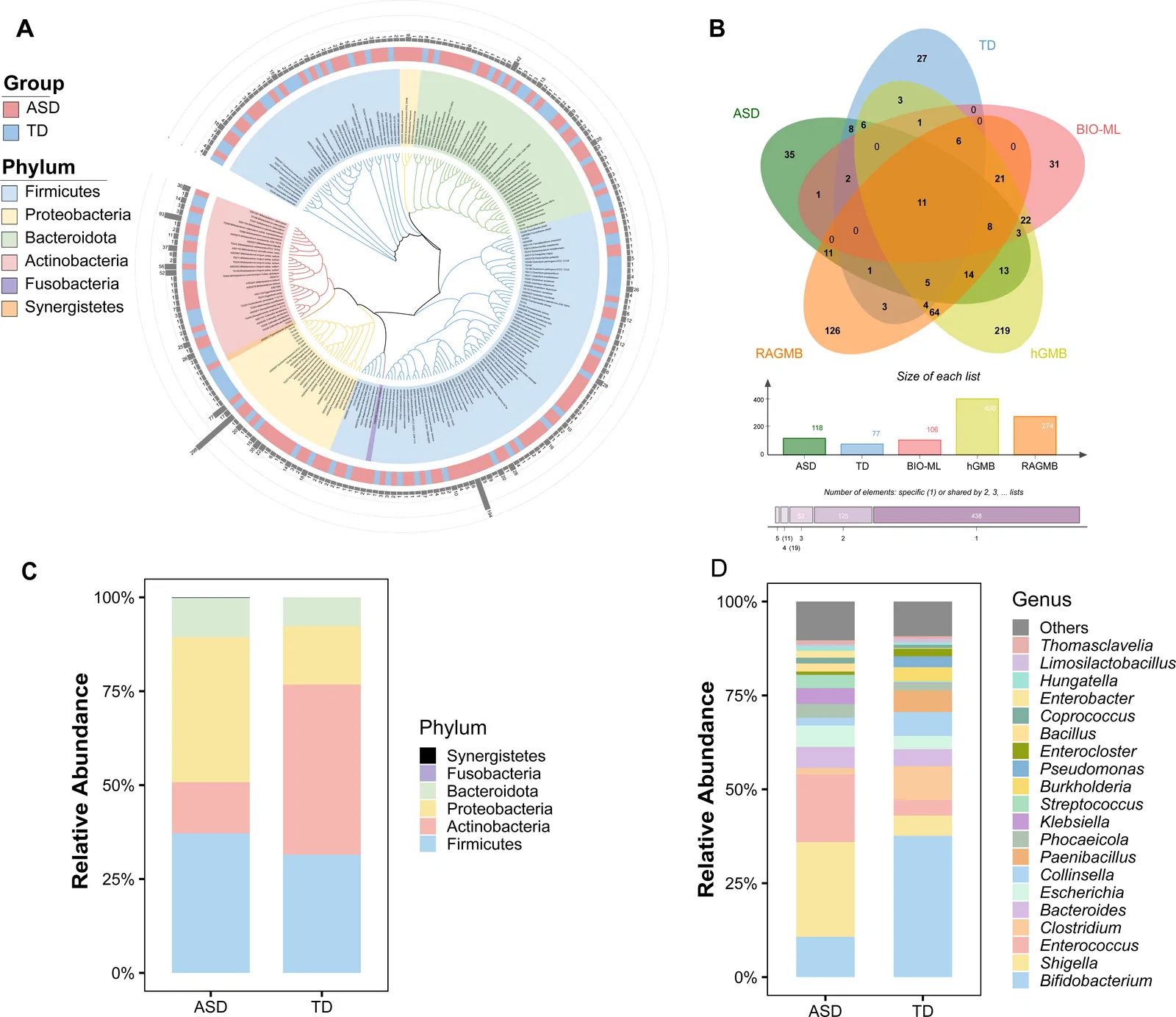
Construction of ASD and TD with bacterial isolation and cultivation. (**A**) The phylogenetic tree shows the non-redundant bacterial strains from fecal samples of the ASD and TD that are coded according to the six phyla. The outer ring bars indicate the number of preserved strains for each cluster. (**B**) The Venn diagram shows the five gut microbial biobanks and overlapping numbers at the species level. (**C**) The relative abundance of ASD and TD at the phylum level. (**D**) The top 20 genera of the relative abundance of ASD and TD.

Many gut microorganisms are difficult to culture in the laboratory. In this study, 1,724 strains were isolated from 41 children with ASD and 12 children with TD using 12 different culture conditions (Table S1). All 1,724 isolates were subjected to 16S rRNA gene sequencing and were classified into six phyla (Firmicutes, Proteobacteria, Actinobacteria, Bacteroidota, Fusobacteria, and Synergistetes) and 72 genera (Fig. 1A). We compared these isolates with strains from three previously reported gut microbiota biobanks, namely hGMB (19), BIO-ML (16), and RAGMB (21), which together contain 780 non-redundant culturable bacterial species. A total of 35 unique intestinal microbiota species were isolated from ASD children and 27 from TD children that were not included in the above three biobanks (Fig. 1B). This expands the currently available human gut bacterial repository with new isolates from the ASD and TD gut microbiota.

We analyzed culturable strains from ASD and TD children. A total of 1,238 bacterial isolates were recovered from 41 ASD children, representing 135 species (Table S2), including 17 novel species (<98.65% sequence similarity [21]), spanning six bacterial phyla. The phyla Proteobacteria (38.61%) and Firmicutes (37.16%) dominated, followed by Actinobacteria, Bacteroidota, Fusobacteria, and Synergistetes (Fig. 1C). Within the ASD bacterial isolates, *Shigella* (25.2%), *Enterococcus* (18.0%), and *Bifidobacterium* (10.7%) were the predominant genera (Fig. 1D). In contrast, 486 bacterial isolates were cultured from 12 TD children, encompassing 97 species (Table S3), including 20 novel species (Table S4), spanning 4 phyla. The phyla Actinobacteria (45.27%) and Firmicutes (31.28%) predominated, followed by Proteobacteria and Bacteroidota (Fig. 1C). In TD isolates, *Bifidobacterium* (37.7%) predominated, followed by *Clostridium* (9.1%) and *Collinsella* (6.4%) (Fig. 1D).

**Fig 2:**
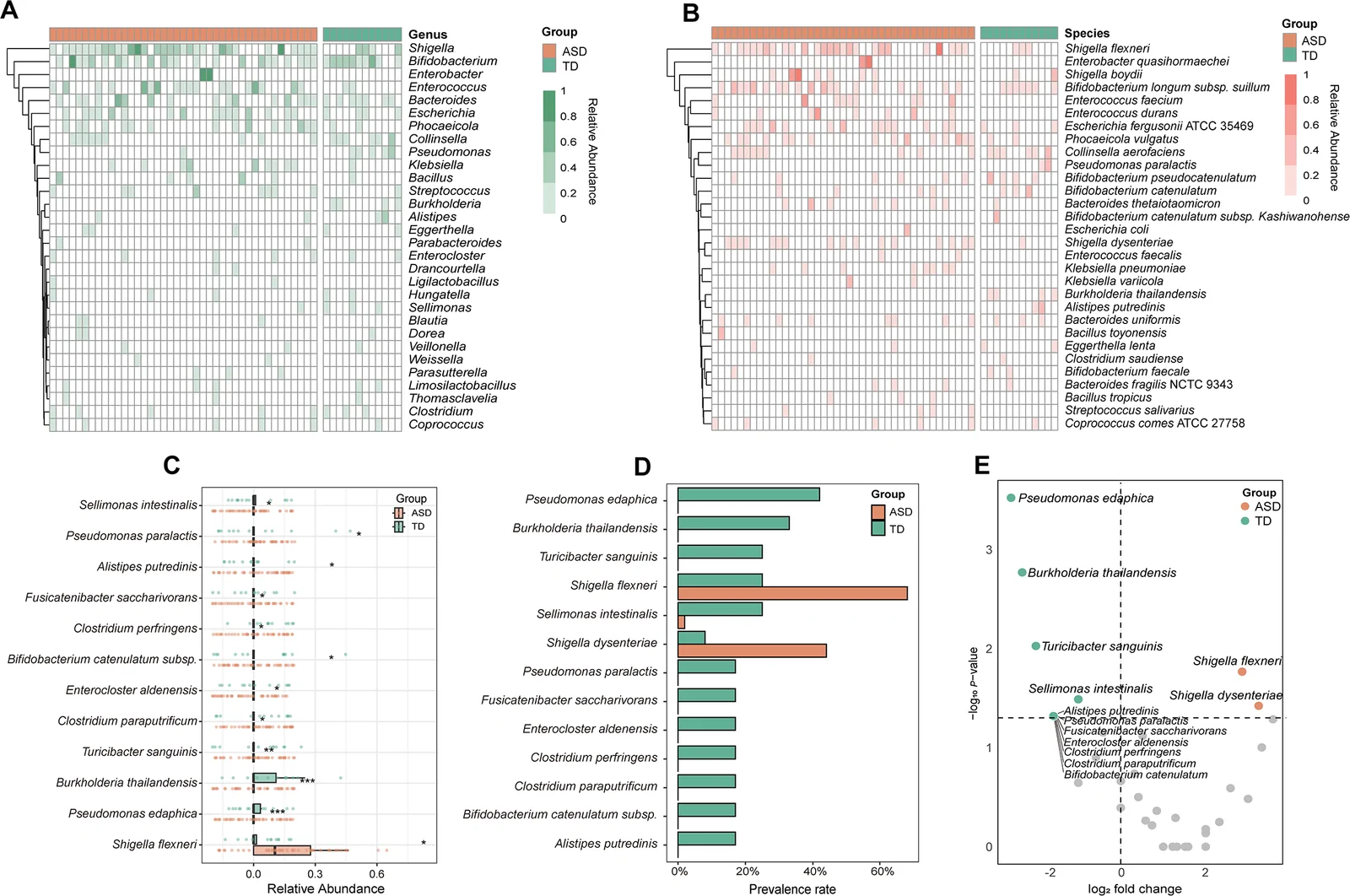
The culturomics reveals microbial composition differences between ASD and TD. (**A and B**) Heatmaps display the relative abundances of the top 30 taxa in the gut microbiota of ASD and TD individuals at the genus (**A**) and species (**B**) levels, respectively. (**C**) Relative abundances of selected bacterial taxa in fecal samples from ASD and TD groups. Boxplots show the distribution of relative abundances for each taxon, with individual points representing individual samples. Group differences were assessed using two-sided Wilcoxon rank-sum tests, followed by Benjamini-Hochberg FDR correction. Asterisks indicate significance levels based on *q* values (*q ≤ 0.2, **q ≤ 0.1, ***q ≤ 0.05). (**D**) Differences in bacterial prevalence between ASD and TD groups. Bars represent the prevalence rate of each taxon in the two groups. Group differences in prevalence were assessed using two-sided Fisher’s exact tests, followed by Benjamini-Hochberg FDR correction. (**E**) A volcano plot represents the statistical significance and fold changes in bacterial prevalence between ASD and TD groups.

The heatmaps (Fig. 2A and B) revealed differential abundance patterns of bacterial species between ASD and TD children in fecal cultures. Several bacterial taxa exhibited differential relative abundance between the ASD and TD groups (Fig. 2C). Using two-sided Wilcoxon rank-sum tests with Benjamini-Hochberg false discovery rate (FDR) correction (*q* ≤ 0.2), multiple taxa showed significant or trend-level differences. Notably, *Pseudomonas edaphica* and *Burkholderia thailandensis* displayed the strongest differences, remaining significant after stringent FDR correction (*q* ≤ 0.05). Additional taxa, including *Turicibacter sanguinis*, *Clostridium perfringens*, *Enterocloster aldenensis*, and *Fusicatenibacter saccharivorans*, demonstrated moderate but consistent differences between groups (0.05 < *q* ≤ 0.2), suggesting potential ASD-associated shifts in gut microbial composition. Specifically, *Shigella flexneri* was cultured in 28 (68%) ASD children versus 3 (25%) TD children. Conversely, *Pseudomonas edaphica* was present in 5 (42%) TD children but absent in ASD children. Statistical analysis of species detection frequencies (Table S5; Fig. 2D) identified significant differences (*q* < 0.05) in the prevalence of specific taxa between groups. Notably, *Pseudomonas edaphica* (*q* = 0.0003) and *Burkholderia thailandensis* (*q* = 0.0017) exhibited significantly higher prevalence rates in TD children compared with ASD children, while *Shigella flexneri* (*q* = 0.0171) and *Shigella dysenteriae* (*q* = 0.0378) showed the opposite pattern. Sensitivity analyses restricted to male participants showed that the relative abundance differences were attenuated and did not fully recapitulate the findings of the primary analysis (Table S6). In contrast, prevalence-based analyses yielded largely consistent results with the main analysis, supporting the robustness of the observed differences in bacterial detection rates (Table S7). The volcano plot (Fig. 2E) further illustrated the magnitude of compositional shifts (log2 Fold Change) and statistical significance (−log_10_ *P* value) across isolated species, reinforcing the above findings. These divergences may indicate potential microbial biomarkers associated with autism and reflect compositional shifts in the gut microbiota of ASD children, warranting further investigation into their mechanistic roles in autism pathogenesis.

### Culturomics elucidates the relationship between clinical outcomes and microbial dynamics during FMT treatment

Of the 41 children with ASD recruited, 17 successfully completed a 9-week oral fecal FMT capsule protocol (Table 2). FMT capsules were administered in weeks 1 and 5. After the ninth week of treatment, participants with autism behavior checklist (ABC) scores below 67 and clinical global impressions scale-improvement (CGI-I) scores of 1–2 were classified into the response group, while others were assigned to the non-response group (22). Ultimately, participants were stratified into response (*n* = 10) and non-response (*n* = 7) groups based on ABC and CGI-I criteria. Meanwhile, fecal samples were collected at baseline, week 5, and week 9, and microbial dynamics during FMT treatment were analyzed by isolation and culture techniques (Table S8). According to group classification, the impact of FMT treatment on the composition of the ASD gut microbiome was further evaluated.

**TABLE 2:** Clinical characteristics of the 17 participants receiving FMT across the three study time points^*a*^

	Baseline	Week 5	Week 9
Gender			
Male	15 (82.3%)		
Female	2 (17.6%)		
Baseline characteristics			
Age	5.82 (1.31)		
BMI	15.79 (1.47)		
Scale score			
ADOS-2	21.78 (3.41)	22.18 (3.40)	22.43 (3.40)
SRS	139.55 (20.88)	137.45 (20.30)	135.67 (20.58)
CARS	34.19 (2.86)	34.03 (2.80)	33.98 (2.77)
ABC	63.47 (27.47)	63.14 (26.81)	64.13 (25.92)
Bristol stool score	3.16 (0.95)	3.26 (0.82)	3.34 (0.79)
GSRS	21.16 (6.83)	21.21 (6.51)	21.07 (6.29)
CGI-I	2.97 (1.09)	2.90 (1.07)	2.80 (0.98)

^*a*^ Data for continuous variables are presented as mean (SD), and categorical variables are presented as *n* (%).

**Fig 3:**
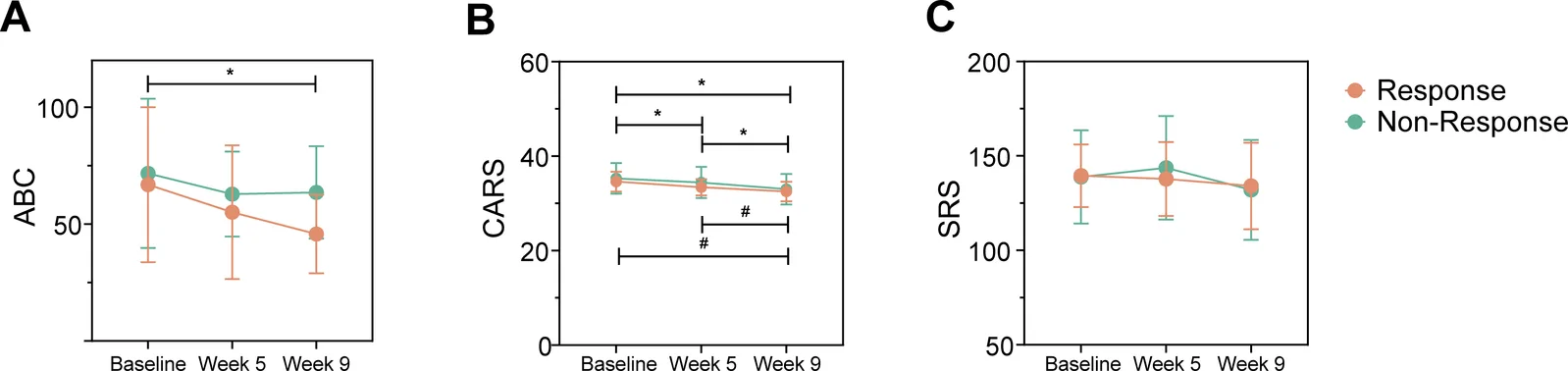
Dynamic changes in clinical scores between response and non-response groups. (**A–C**) Dynamic changes in ABC (**A**), CARS (**B**), and SRS (**C**) scale scores of the responder and non-response groups across the three time points. Two-way ANOVA was used to evaluate group and time effects, and post hoc multiple comparisons were conducted with Benjamini-Hochberg FDR correction. * Response group, *q* < 0.05; # non-response group, *q* < 0.05.

The scores of the response and non-response groups on the social responsiveness scale (SRS), childhood autism rating scale (CARS), and ABC scales are shown in Fig. 3A through C, with group and time effects evaluated using two-way analysis of variance (ANOVA), followed by multiple comparisons analysis. The results demonstrated that the response group exhibited significantly lower ABC (*q* < 0.01) and CARS (*q* < 0.01) scores at post-FMT week 9 compared with baseline. In contrast, the non-response group showed no significant changes in ABC and SRS scores, although CARS scores (*q* < 0.01) were significantly reduced post-treatment at week 9.

### Longitudinal culturomics analysis of gut microbiota in ASD responders to FMT

**Fig 4:**
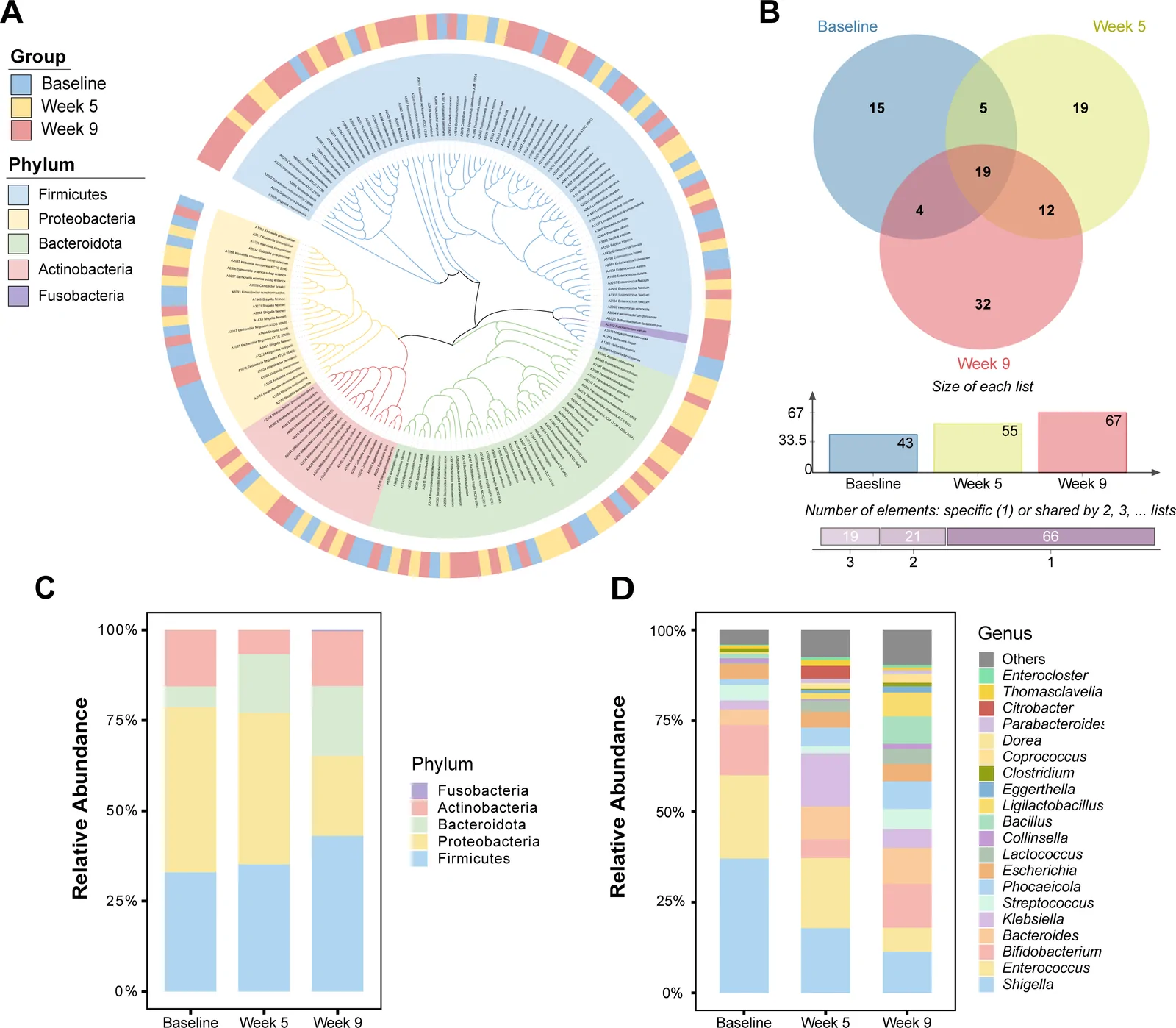
The response group stool microbiota changes with FMT. (**A**) The phylogenetic tree shows the classification diversity of the response group with the FMT. (**B**) The Venn diagram displays the unique and shared classified strains of the response group at the three time points. (**C**) The relative abundance at the phylum level of the response group at the three time points. (**D**) The top 20 genera of the relative abundance of the response group at the three time points.

To better understand the impact of FMT on the bacterial diversity of the gut microbiome in the ASD cohort, we further analyzed bacterial strains in both the response and non-response groups (Fig. S1). Since patients in the non-responsive group for ASD did not respond to FMT treatment, we focused on describing the gut bacteria of the responsive group. All 940 isolates from the response group were sequenced for 16S rRNA genes and were classified into five phyla: Proteobacteria (37.3%), Firmicutes (36.7%), Actinobacteria (13.1%), Bacteroidota (12.8%), and Fusobacteria (0.1%). We analyzed strains from three time points in the response group. Based on 16S rRNA gene sequences, we constructed non-redundant phylogenetic trees for the response group (Fig. 4A), revealing distinct clustering patterns at different phyla. Then, we identified a total of 165 bacterial strains in the response group, including both shared and unique strains across the three time points. Notably, the number of unique strains increased at week 9 following FMT, suggesting a dynamic shift in the cultivable microbiota during treatment (Fig. 4B). Furthermore, during the course of FMT, the relative abundances of the phyla Firmicutes and Bacteroidota increased, while that of Proteobacteria decreased (Fig. 4C). FMT treatment led to an increase in the diversity of gut bacteria genera in the response group, with significant differences in the community at the genus level by week 9 compared with baseline (Fig. 4D). The genus relative abundance of *Bacteroides*, *Phocaeicola*, *Lactococcus,* and *Ligilactobacillus,* which include certain beneficial bacteria (23–25), increased by 5.7%, 6.1%, 3.8%, and 6.1%, respectively, at week 9 compared with baseline. Conversely, the relative abundance of *Shigella* and *Enterococcus* decreased by week 9 compared with baseline.

### Dynamic changes in cultivable gut microbiota following FMT in children with ASD

**Fig 5:**
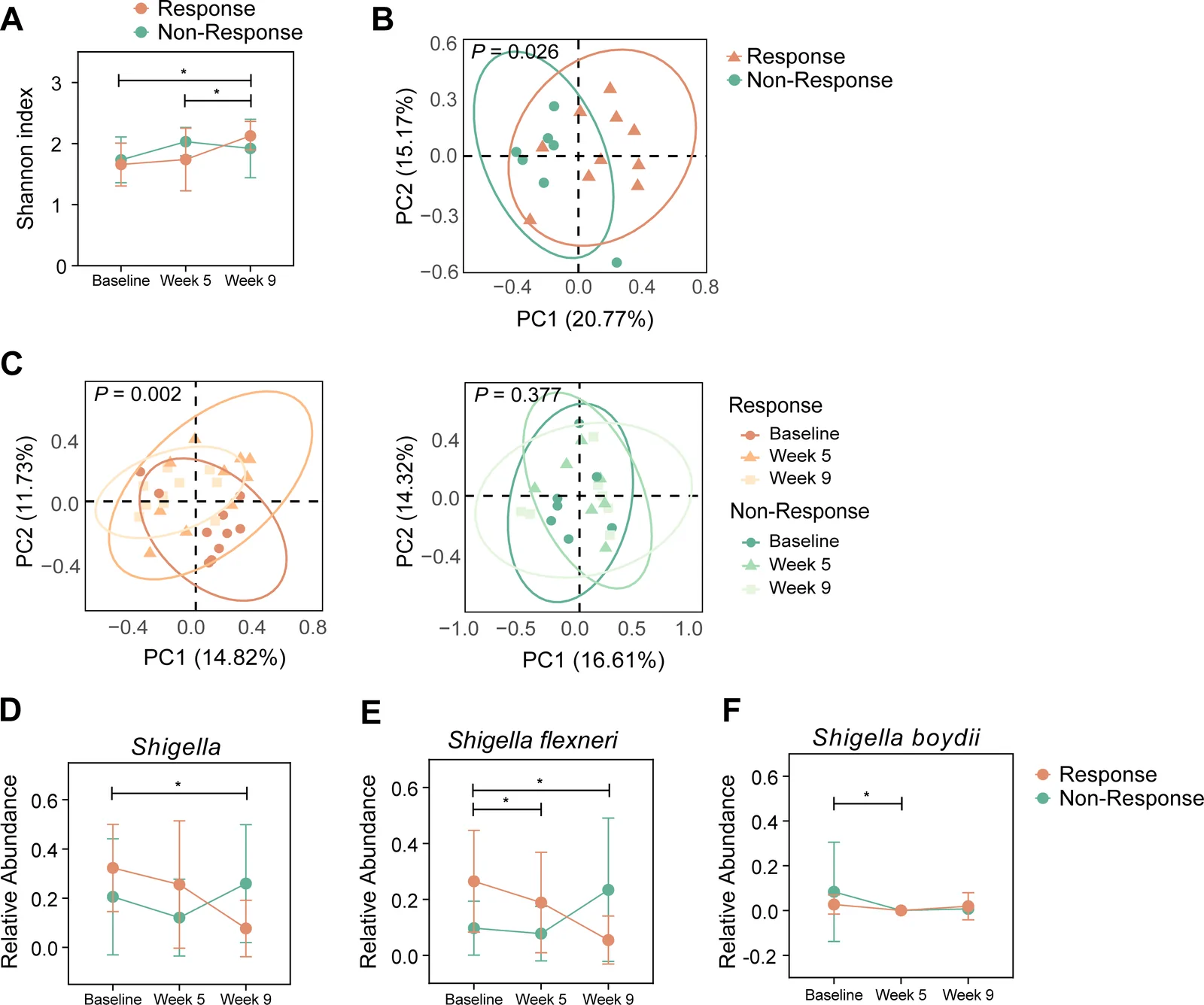
Dynamic changes in microbial diversity between response and non-response groups. (**A**) Shannon index. (**B**) PCoA plot at baseline for the response and non-response groups. (**C**) PCoA plots for three time points for the response group and the non-response group, respectively. (**D–F**) The dynamic changes of *Shigella* (**D**), *Shigella flexneri* (**E**), and *Shigella boydii* (**F**) across the three time points. * Response group, *q* < 0.05.

Further analyses were conducted to assess changes in microbial diversity of children in the response and non-response groups during the treatment. As treatment progressed, the Shannon index increased significantly over time in the response group after FDR-adjusted two-way ANOVA (Fig. 5A), whereas no significant changes were observed in the non-response group. Principal coordinate analysis (PCoA) based on the Bray-Curtis distance revealed a significant difference between the response and non-response groups at baseline (*P* < 0.05) (Fig. 5B). With the treatment of FMT, comparison between baseline and post-treatment periods revealed a significant change (*P* < 0.05) in the community structure of the response group, whereas no difference was observed in the non-response group (Fig. 5C). Further examination of microbial changes revealed a significant reduction in the relative abundance of the genus *Shigella* over the course of treatment in the response group, with a significant difference observed between baseline and week 9 based on two-way ANOVA, followed by Tukey’s multiple comparisons test (Fig. 5D). In contrast, no significant temporal changes were detected in the non-response group. We further analyzed the genus *Shigella* and found that both *Shigella flexneri* (*q* < 0.05) and *Shigella boydii* (*q* < 0.05) were significantly reduced in the response group but remained unchanged in the non-response group (Fig. 5E and F). Notably, earlier findings from this study indicated that children with ASD exhibited a significantly higher prevalence of *Shigella flexneri* in their feces compared with TD controls. These results suggest that FMT not only enhances alpha diversity in the response group but also positively modulates microbiome composition by reducing specific pathogenic bacteria.

We have previously demonstrated that FMT effectively ameliorates behavioral abnormalities in the response group of patients with ASD and significantly reduces the prevalence of harmful intestinal bacteria. Building on this finding, the present section further investigates which potential beneficial bacteria are selectively retained in the FMT-responsive ASD group. Bacterial strains were compared between the ASD and TD groups, identifying 24 strains shared by both groups and 59 unique species specific to the TD group (Fig. S2A). Subsequent analysis compared species from ASD patients in the week 5 and week 9 response phases with TD-unique species. Dynamic monitoring revealed a notable increase in the number of such shared strains across treatment stages: only two shared strains were identified in the week 5 response phase (*Bacteroides fragilis* NCTC 9343 and *Enterococcus hulanensis*), whereas this number significantly rose to nine in the week 9 response phase (*Bacteroides fragilis* NCTC 9343, *Anaerostipes hadrus*, *Parabacteroides merdae*, *Ruthenibacterium lactatiformans*, *Bacteroides salyersiae*, *Clostridium perfringens* ATCC 13124, *Enterocloster lavalensis*, *Turicibacter sanguinis*, and *Weissella confusa*) (Fig. S2B). This gradual increase in ASD-TD shared strains suggests that FMT may drive the gut microbiota of ASD patients toward a composition more similar to that of TD individuals, which could be associated with the therapeutic effects of FMT on ASD symptoms.

## DISCUSSION

Research on the gut microbiota in ASD has grown rapidly. Previous 16S rRNA gene sequencing and metagenomic studies have described community-wide compositional and functional alterations, including gut microbial dysbiosis, delayed microbial maturation, and microbial signatures with potential diagnostic relevance (26–29). However, sequencing-based approaches do not provide live isolates and therefore cannot directly support strain-level functional validation. In contrast, culturomics enables the recovery of live bacteria for experimental investigation. Here, we established an ASD-focused culturable microbiota resource by isolating and preserving 1,724 bacterial strains. This contribution goes beyond complementing sequencing-based observations because it provides an experimentally tractable strain-level resource for downstream functional studies. This is particularly valuable in the context of FMT, where intervention-associated microbial shifts help prioritize candidate taxa for mechanistic follow-up. At the same time, culturomics is selective by nature and reflects only the cultivable fraction of the gut microbiota rather than the full community or true *in vivo* relative abundance (30). Future studies integrating culturomics with metagenomic approaches will therefore be important for linking strain-level variation to functional and mechanistic insights.

A notable finding of this study is the enrichment of *Shigella flexneri* and *Shigella boydii* in the cultivable gut microbiota of children with ASD. Functionally, emerging evidence has demonstrated that *Shigella* species can invade intestinal epithelial cells, impair intestinal barrier integrity, and trigger local intestinal inflammation (31, 32). Meanwhile, we found that the relative abundance of *Shigella* was significantly decreased over the course of treatment in the response group. Combined with these findings, we propose a working hypothesis that *Shigella* enrichment may mark a gut ecosystem state associated with barrier dysfunction and inflammatory signaling in a subset of children with ASD. Notably, the causal relationship and underlying mechanisms remain unvalidated in the current study, and further mechanistic experiments are required for verification. Our observations are broadly consistent with previous 16S and metagenomic studies showing gut microbial disturbances in ASD, including enrichment of Proteobacteria-related signals and associations with taxa, such as *Clostridium*, although taxonomic findings vary across cohorts (11, 33). Reviews and systematic summaries have likewise emphasized substantial heterogeneity across ASD microbiome studies, likely reflecting differences in age, geography, diet, gastrointestinal symptoms, analytical platforms, and bioinformatic pipelines (34–36). Against this background, culturomics exhibits unique advantages, as it converts association signals into culturable candidate strains for subsequent mechanistic investigation.

In addition to reducing harmful bacteria, FMT responders showed enrichment of beneficial strains. Species such as *Bacteroides fragilis* (37), *Parabacteroides merdae* (38–40), *Bacteroides salyersiae* (41–43), *Anaerostipes hadrus* (44, 45), *Ruthenibacterium lactatiformans* (46), *Turicibacter sanguinis* (47, 48), and *Weissella confusa* (49). These taxa are known to produce short-chain fatty acids, regulate bile acid metabolism, and reduce oxidative stress. Such functions can support intestinal homeostasis, modulate host immunity, and exert neuroprotective effects. Importantly, many of these beneficial taxa were also enriched in TD children, suggesting that FMT was associated with the microbial community of ASD responders toward a more “TD-like” state. The gradual acquisition of TD-enriched strains during treatment, coupled with clinical improvement, highlights species-level microbiota remodeling as a key mechanism underlying FMT efficacy. This finding is particularly meaningful from a culturomics perspective because it identifies concrete candidate strains for follow-up validation rather than only reporting abstract compositional shifts.

Our study underscores the influence of baseline gut microbiota on FMT outcomes in children with ASD. Notably, responders and non-responders showed distinct pre-treatment microbial profiles, with *Shigella* abundance emerging as a potential predictor of therapeutic success. After FMT, responders not only exhibited partial symptomatic improvements but also showed increased microbial diversity and reduced levels of *Shigella flexneri* and *Shigella boydii*, suggesting that treatment reshaped the gut ecosystem by promoting beneficial taxa while suppressing potential pathogens. These findings support the involvement of the gut-brain axis in ASD and highlight the promise of using pre-treatment microbial signatures to guide patient stratification and personalized interventions (50). Inter-study variations, however, remind us that host factors, donor characteristics, and treatment protocols can all influence outcomes (51–53).

Despite these promising findings, our study has some limitations. The number of children completing longitudinal FMT profiling was modest, and larger trials will be needed to validate our findings. Functional experiments using isolated strains are also essential to confirm their mechanistic roles. In addition, because culturomics depends on selective growth conditions, some gut microorganisms may not have been recovered, and the cultured profiles reported here do not necessarily reflect absolute or relative abundance in the original fecal community. The taxonomic assignments in this study were primarily based on 16S rRNA gene sequencing, which may have limited resolution for distinguishing closely related organisms; therefore, future whole-genome sequencing of key isolates will be important, particularly for *Shigella*-related strains. Nevertheless, these limitations do not diminish the central contribution of the study: we provide an ASD-focused, strain-level culturable resource together with longitudinal evidence of microbiota remodeling during FMT, thereby establishing an experimentally actionable framework for moving from association to mechanism.

### Conclusions

In summary, we used culturomics to generate an ASD-focused strain-level gut microbial resource and to characterize microbial dynamics during FMT in children with ASD. We identified *Shigella flexneri* and *Shigella boydii* as candidate ASD-associated taxa that were more frequently recovered in ASD samples and decreased in FMT responders, while TD-enriched beneficial strains emerged during clinical improvement. These findings address a key limitation of sequencing-based studies by providing live isolates for direct functional validation. Future work should build on this resource to determine which cultured strains act as microbial markers, mechanistic drivers, or therapeutic mediators in ASD.

## MATERIALS AND METHODS

### Study participants

In this study, we recruited 41 children with autism from Jinan Children’s Hospital according to certain inclusion criteria, while 12 children with TD were recruited as controls. All participants of the autism group were diagnosed with ASD using the Autism Diagnostic Observation Schedule, second edition (ADOS-2). Prior to recruitment, information regarding the previous two months of antibiotic treatment, clinical characteristics, for example, BMI, was obtained from each child, alongside a record of the ASD activity score and other relevant data.

The exclusion criteria for the study were as follows: (i) secondary autism caused by identifiable factors, such as metabolic abnormalities or genetic diseases, (ii) serious gastrointestinal conditions that required immediate treatment, (iii) severe immunodeficiency, (iv) severe malnutrition or underweight, and (v) children undergoing special dietary therapy.

### FMT intervention

Of the 41 children with ASD enrolled in this study, 17 consented to receive FMT and completed the 9-week study with good compliance. The study period consisted of a 5-week FMT treatment phase, followed by a 4-week follow-up observation period. Before FMT treatment, all participants underwent capsule-swallowing training to ensure that they were able to take the oral capsules as required. To prepare for FMT, all participants followed a low-residue semi-liquid diet for 3 days before treatment to facilitate bowel cleansing and reduce gastrointestinal motility, thereby promoting colonization of the transplanted microbiota. In addition, GOLYTELY (polyethylene glycol) was administered 1 day before FMT to ensure bowel evacuation. Oral FMT capsules were administered during week 1 and again during week 5. Specifically, participants received eight capsules in the morning and eight capsules in the evening each day for six consecutive days during each treatment course. After the 5-week treatment phase, participants were further monitored for an additional 4 weeks. Throughout the FMT program, all participants continued to receive their routine rehabilitation training.

Healthy stool donors were screened based on international expert consensus (54–58). Complete blood count, liver and kidney function tests, electrolyte levels, and C-reactive protein were all within normal ranges. Screening for infectious pathogens was negative, and routine stool examination, including testing for *Clostridium difficile*, revealed no abnormalities. The donor’s psychological status was evaluated as normal based on multiple psychological scales and assessment by qualified clinicians. No abnormalities were identified in the donor’s past medical history or personal history.

The fecal microbiota capsules were produced according to the international guidelines (57, 59). Briefly, donor stool samples were collected in sterile, contaminant-free containers and transported to the laboratory at 4°C–8°C within 1 h. In a cleanroom facility, each sample was processed individually under anaerobic and aseptic conditions. Qualified stool specimens were weighed, aliquoted for storage at −80°C, homogenized with chilled sterile saline, and sequentially filtered to remove large particulate and fibrous material. The resulting suspension was then centrifuged, resuspended in cryoprotective agents, lyophilized, and packed into acid-resistant hypromellose capsules. The finished capsules were stored at −80°C until use.

### Sample collection and treatment

Fecal samples from children with ASD and TD were collected at Qilu Hospital of Shandong University. Of the 41 ASD children enrolled, 17 consented to subsequent FMT treatment and exhibited good compliance. A 9-week FMT intervention and follow-up period was administered to these 17 ASD children, with fecal samples collected at baseline, week 5, and week 9, and subjected to cultivation and isolation to monitor microbial dynamics during treatment. In total, 87 qualified fecal samples were gathered from all participants.

Each fecal sample was divided into two portions: one was immediately stored at −80°C for DNA extraction, while the other was placed in a vertical anaerobic culture bag (Hopebio, China) containing AneroPack-Anaero (MGC, Japan) and an anaerobic indicator (MGC, Japan), maintained at 4°C.

This sample was transferred to an anaerobic workstation (LONGYUE, China) within 4 h for gut microbiome isolation, where the gas composition consisted of 90% N_2_, 5% CO_2_, and 5% H_2_.

### Bacterial isolation and cultivation

Fresh feces samples were suspended in an equal volume of phosphate-buffered saline and vigorously vortexed. The suspension was filtered through a cell strainer to remove large insoluble particles in the suspension and then serially diluted from 10^−1^ to 10^−7^. Subsequently, 100 μL of the dilution of 10^−6^ and 10^−7^ were spread on different agar plates, followed by anaerobic incubation at 37°C. Twelve distinct culture conditions and media formulations were used for bacterial cultivation (Table S1).

Isolation and identification of colonies were carried out as follows: single colonies were picked from agar plates incubated for 3–30 days, and then placed in the corresponding liquid medium and incubated under the same culture conditions for the same duration as the initial incubation period.

### 16S rRNA gene sequencing

Subsequently, an appropriate volume of bacterial suspension was aliquoted and subjected to PCR amplification of the 16S rRNA gene using 2× Rapid Taq Plus Master Mix (Dye Plus) (Vazyme, China) and universal primers for the 16S rRNA gene (primers: 27F: 5′-AGAGTTTGATCCTGGCTCAG-3′; 1492R: 5′-GGTTACCTTGTTACGACTT-3′). The PCR products were sequenced using Sanger sequencing (Sangon Biotech Ltd., China).

The sequencing results were analyzed using BLAST against the NCBI 16S ribosomal RNA sequence database (last updated on 27 August 2024) to classify the strains. Based on the 16S rRNA gene sequence identity, with a threshold of 98.65% for distinguishing different species, both corresponding species and potential novel species of the strains were identified. A strain was classified as an existing species if its 16S rRNA gene sequence identity with all validly published species in the databases exceeded 98.65%. Conversely, if this identity was 98.65% or less, the strain was considered a potential novel species.

The non-redundant set of 16S rRNA gene sequences was clustered using CD-HIT version 4.8 with a sequence identity of 0.9865 (60). The phylogenetic relationship between isolates was determined by aligning the non-redundant set of 16S rRNA gene sequences to construct a maximum-likelihood tree by using FastTree version 2.1.11. After construction, the tree was edited using the Interactive Tree of Life website (61).

### The judgment basis of different strains and the preservation strategy

We sorted out the source of the isolates and the 16S rRNA gene sequencing results. Isolates were considered to be different strains when the 16S rRNA gene similarity between the two strains was less than 98.65%.

All strains were preserved in the following way: cultures were grown on the corresponding agar media until visible single colonies appeared. Single colonies were picked and cultured in the corresponding liquid medium. Cultures were grown to late logarithmic phase under appropriate growth conditions. Then, 0.8 mL bacterial culture was mixed with 0.8 mL of 60% glycerol and stored in frozen-storage tubes. The freeze-storage tubes were stored in the refrigerator at −80°C.

### Statistical analysis and visualization of the results

All statistical analyses and data visualization were performed using GraphPad Prism 10.1 and R software (version 4.3.3). Gut microbiota profiles were compared between individuals with ASD and TD controls. Differences in relative abundance of bacterial taxa between ASD and TD groups were assessed using two-sided Wilcoxon rank-sum tests, with *P* values adjusted for multiple comparisons using the Benjamini-Hochberg FDR procedure. Differences in bacterial prevalence between groups were evaluated using two-sided Fisher’s exact tests with FDR correction.

Longitudinal changes in clinical scores (ABC, CARS, and SRS) and microbial diversity indices were analyzed using two-way ANOVA, followed by post hoc multiple-comparisons tests (Tukey’s test or FDR-adjusted comparisons, as specified for each analysis). Beta diversity was assessed using PCoA based on Bray-Curtis dissimilarities, and group differences were tested using permutational multivariate analysis of variance (PERMANOVA). Sensitivity analyses were performed by restricting comparisons to male participants to evaluate the robustness of ASD-TD differences. Unless otherwise specified, statistical significance was defined as *P* < 0.05.

## Data Availability

The 16S rRNA sequences and annotation files for all cultured strains are publicly available on Zenodo (https://doi.org/10.5281/zenodo.19738247).

## SUPPLEMENTAL MATERIAL

Supplemental Figures spectrum.00797-26-s0001.docx Figures S1 and S2.

Supplemental Tables spectrum.00797-26-s0002.xlsx Tables S1 to S8.

ASM does not own the copyrights to Supplemental Material that may be linked to, or accessed through, an article. The authors have granted ASM a non-exclusive, world-wide license to publish the Supplemental Material files. Please contact the corresponding author directly for reuse.
