# Supplementary material for: Gut microbial culturomics identifies autism-associated Shigella and reveals species-level remodeling during fecal microbiota transplantation
Source: Microbiol Spectr. 2026 Jun 4;14(7):e00797-26. doi: 10.1128/spectrum.00797-26 (PMC13339971; doi:10.1128/spectrum.00797-26)
Supplement: Supplemental Figures — Figures S1 and S2. [file spectrum.00797-26-s0001.docx]

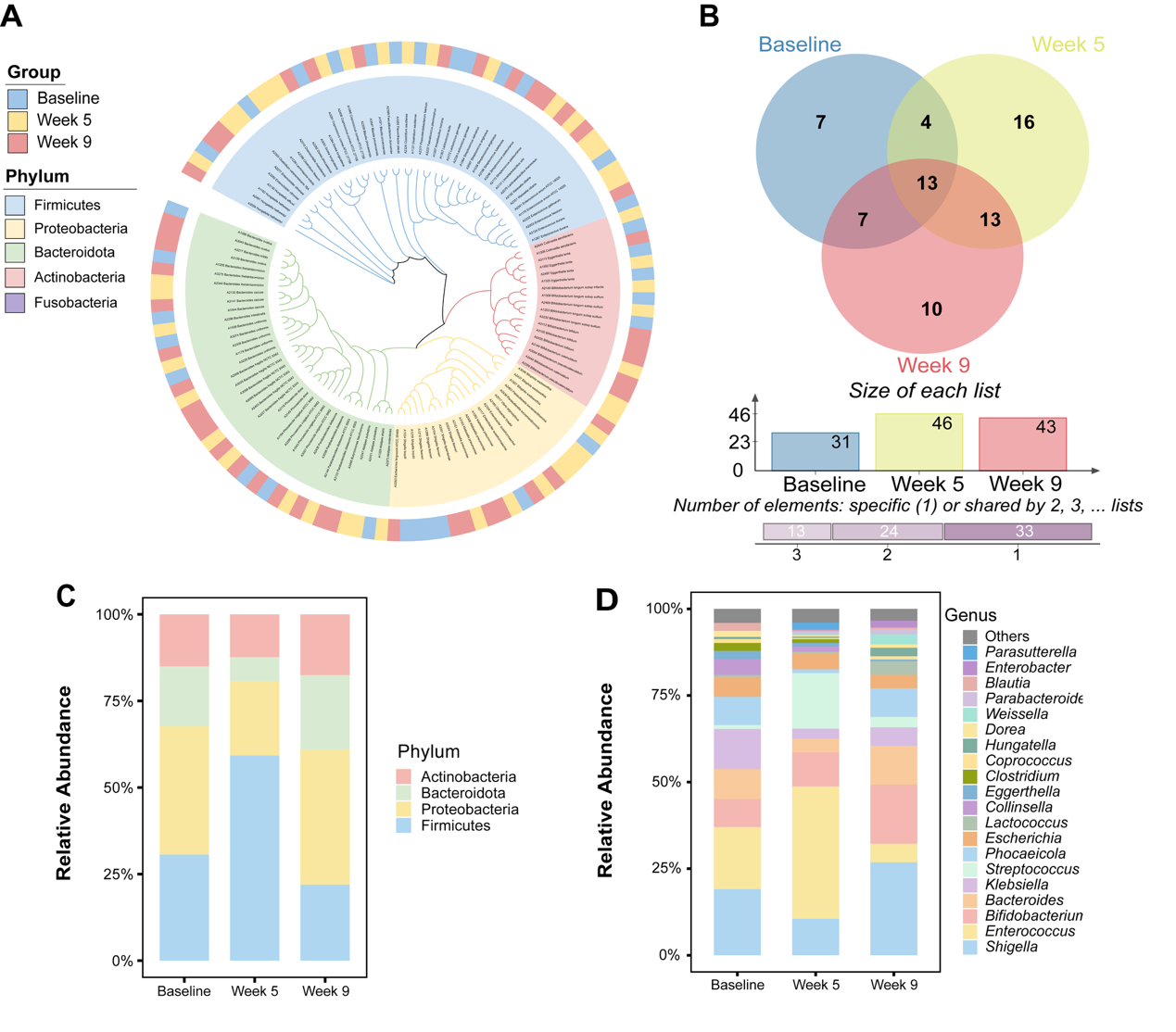


**Figure.S1 Stool microbiota changes with FMT in the non-response group**

(A) The phylogenetic tree shows the classification diversity of the non-response group with the FMT. (B) The Venn diagram displays the unique and shared classified strains of the non-response group in the three time points. (C) The relative abundance at the phylum level of the non-response group in the three time points. (D) The top 20 genera of the relative abundance of the non-response group in the three time points.


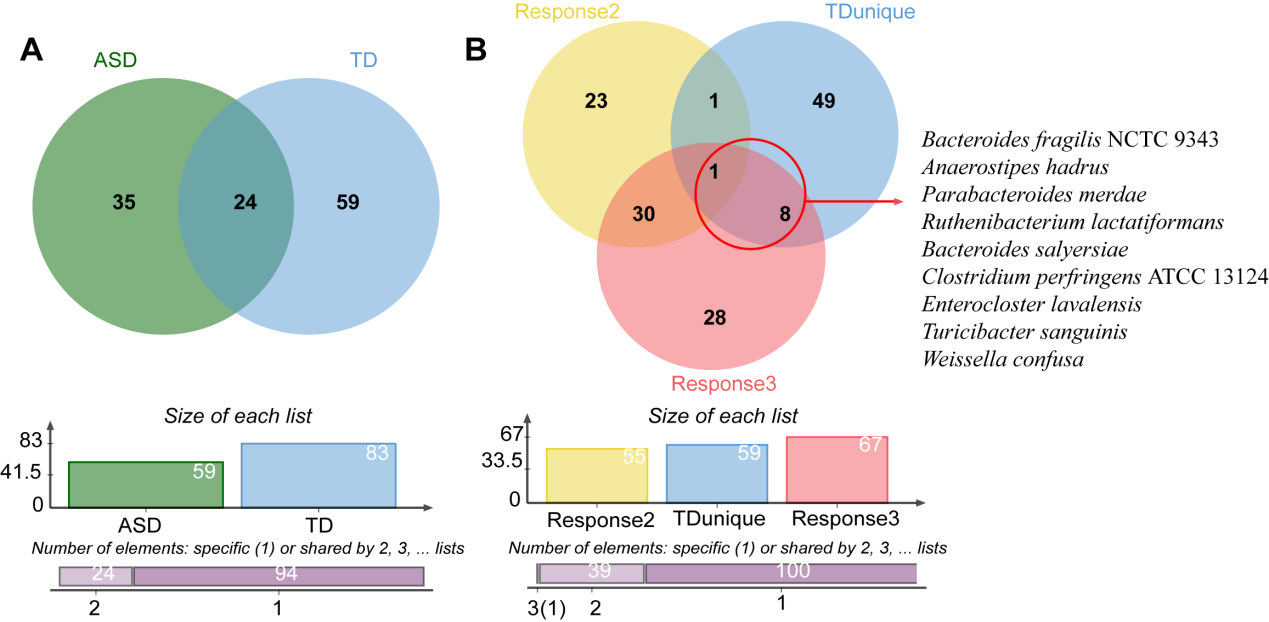


**Figure.S2 Increased gut bacteria of Children in the ASD response group during FMT treatment.**

(A) Bacterial strains were compared between the ASD and TD groups, identifying 24 strains shared by both groups and 59 unique species specific to the TD group.

(B) Comparing ASD response group species at weeks 5 and 9 with TD-specific species revealed two shared species in the week 5 response group and nine shared species in the week 9 response group. Response2 = Response group at week 5; Response3 = Response group at week 9.
